# Preventive Orthodontia in Early Childhood and the Management of Children

**Published:** 1919-05

**Authors:** Herbert A. Pullen

**Affiliations:** Buffalo, N. Y.


### PREVENTIVE ORTHODONTIA IN EARLY CHILDHOOD AND THE MANAGEMENT OF CHILDREN

HERBERT A. PULLEN, D.M.D., BUFFALO, N. Y.

Looking backward from the present day practice of early treatment of malocclusion, it is a far cry to the days of almost a quarter of a century ago, when the patients of the few orthodontic specialists consisted entirely of adults. The diagnosis and treatment of developing malocclusions in children had hardly been touched upon at that time, and the common advice of the dentist to the parents of children afflicted with malocclusions was to "wait until the permanent teeth were all erupted" before any corrective methods were started, a sparring for time piece of advice that at least afforded the dentist who proffered it a chance to temporarily evade the responsibility of an intelligent decision in regard to a case, and, if the dentist was beyond middle age when he gave the advice, he would in all probability have one foot in the grave and be incapacitated for active practice when the patient, grown up, and with the permanent teeth all erupted should reappear for the treatment of the mal occlusion.

Of a similar purport with intent to shirk responsibility by procrastination as a shield for ignorance is the advice to "let Nature alone and she will correct the malocclusion," with the result in most cases of the development of a serious malocclusion confirmed by long years of arrested or deficient growth of the dental arches, while the child is allowed to patiently wait without avail for Nature to begin her corrective operations.

Another fallacy of early dental origin is the theory of "extraction to make room" in the dental arch for teeth which are out of alignment. The advice thus freely given seems to have been handed down or inherited from one generation of dentists and their patients to another, so that, even at the present day, it not infrequently crops out in unthinking repetition of time-worn and obsolete phrases such as have been quoted.

In order to contradict this erroneous advice it is necessary to substitute certain facts gained from long orthodontic experience for the fallacious theories presented at the beginning of this essay. To begin with, it must have occurred to the most casual observer of today that the chief aim of both medical science and its branch, dental science, has changed from curative to preventive methods of treatment.

If this aim is to be carried out consistently, it follows that in order to prevent disease or deformity the beginning of all preventive and prophylactic measures must be directed upon the human organism from the time of its earliest infancy. "From the cradle to the grave" has now become the motto of the health hygienist, and thus it is that the child, and its environment, its work and its play, but chiefly its health, is the center of all absorbing interest in all branches of medicine, especially in orthodontia.

The ideal physical condition which we all have in our minds as possible for the child is represented by such a state of normality of bodily function and structure that there is no room left for disease.

This mental ideal of the normal, physically perfect child encompasses health, beauty, and happiness. When one considers the charm of a well-proportioned and well-balanced face, with a winning smile, and displaying a beautiful row of pearly teeth, the mental picture becomes extremely fascinating.

Evidences of faulty respiration or of undeveloped and asymmetrical dental arches are inconceivable in the perfectly normal face; in fact, the whole external face reflects the evidence of an anatomically perfect internal face, with well developed dental arches, well developed nasal passages, and a healthy tonsillar ring. A step farther, and we may read as well a normally healthy body in its entirety, and a healthy mind, the expression of which is complete and care-free happiness.

Every mother desires such a delightful combination of health, beauty, and happiness for her children, and is willing to undergo any sacrifice to obtain it, but many mothers are doomed to disappointment, for either through hereditary influence or some acquired physical defects, the consummation of ideal physical perfection and beauty seems impossible in child development in many cases, except under peculiarly advantageous circumstances.

Beauty of the face is a composite of normally developed, well balanced features, and no one part of the face lends more attractiveness to this composite picture than perfectly developed teeth and dental arches, and no other part of the face can so deform and make ugly the lines of an otherwise beautiful face as the crooked, irregularly placed, or protruded teeth so often noticed in the mouths of children.

Take as an illustration a child exhibiting a malocclusion which is extreme in its manifestations of oral and facial deformity, and hardly amenable to corrective treatment because of the profound disturbance of bodily functions as a whole, a condition belonging to a type of cases which are occasionally observed in practice. The facial configuration of such a case is pathetically distressing with its open,. drooping mouth, protruding teeth, and inharmonious facial lines, exhibiting a typical dullness of expression.

In this type of case the nasal passages are undeveloped, the nasal septum deflected, the turbinates enlarged, adenoids and enlarged tonsils are in evidence, the hearing is impaired and there is a muffled tone to the voice. Further examination of such a child usually reveals a narrow chest, and consequent lack of respiratory function, a stunting of growth and underweight, all evidences of a hard struggle for existence.

This is not an infrequent history, though in some cases it is perhaps less marked, of many of the children who are suffering from what has been aptly called the "vicious cycle" in the oral and nasal cavities.

However successfully, the rhinologist can remove adenoids, tonsils and nasal obstructions for the restoration of normal nasal respiration and ventilation and drainage of the nasal sinuses, and the ophthalmologist relieve eye strain and assist in the proper correlation of eye functions, there still remains a further field of operations in orthodontia, which, because of its possibilities of assisting in the development of the internal and external face, because of its stimulation to bone building in the maxillae and superimposed structures of the nose, and its consequent restoration of the functions of nasal respiration and normal occlusion, bids fair to fill an unique and highly important place in its possibilities of further physical benefit to the child.

But at this point it should be emphasized that the greatest benefit from orthodontic treatment is obtained in the early years of childhood, when such pathological changes as arrested development of the maxillae of the nasal structures are first apparent, and before these conditions are confirmed by several years of mouth breathing with its many ills, and before abnormal development of the internal and external structures of the face have shut the door to the best opportunity for effective treatment.

With added years the long continued effect of faulty respiration and lack of full function of the organs of the internal face, because of nasal obstruction, mouth breathing and malocclusion of the teeth results in the extreme of abnormal conditions in adult life. The lines of the face become sadly out of balance, the teeth are protruded, and the chest often is caved in. Such a case is manifestly too mature for ideal treatment, for although improvement in occlusion might be obtained, the bone is too dense for development of the dental arches and superimposed nasal structures, and it is obvious that the patient has waited too long before commencing corrective treatment.

However, in the treatment of a young child having the severe type of malocclusion associated with respiratory troubles, very pleasing results may be obtained in dental arch development, in better breathing conditions, a relief from the dull expression of mental hebetude, and the restoration of beauty to the face.

It must be evident to the close observer that malpositions of the teeth, generally speaking, are but objective symptoms of abnormal development of the dental and maxillary arches, usually exhibited in an arrest or deficiency of development, such as contracted arches with not enough room for the permanent teeth to erupt without crowding some of them out of alignment. In a simple case of deficient development of the dental arches, there is crowding of the teeth and overlapping in their effort to erupt in dental arches which are too small for them. A consistent corrective treatment should be begun during the growing age of childhood when a very pleasing result in the development of the dental arches and restoration of normal occlusal relations may be obtained.

Whether or not the general practitioner of dentistry ever practices orthodontia, it is essential for his professional reputation that he does not err in his advice to parents regarding the incipient malocclusions in their children's mouths, but he should either institute corrective measures himself in these cases or refer them to the orthodontic specialist as early as the malocclusion becomes evident.

The time when corrective treatment should be begun has somewhat definitely arranged itself. Since the roots of the deciduous molars enclose the crowns of the permanent bicuspids, any lateral pressure exerted in the expansion of the deciduous arch is transmitted to the permanent bicuspids, and it is therefore advisable to begin operations for the expansion of the deciduous dental arch some little time before the loosening of the deciduous molars preparatory to their being shed, or else the looseness or absence of these deciduous teeth will postpone any attempts at arch expansion until the full eruption of the bicuspids, some time later.

Thus, in the anterior development of a lower dental arch, begun at the age of eight years, the deciduous cuspids and molars are intact and their roots have not begun to absorb, so that they afford a firm support for the appliances which may be adjusted for anterior arch expansion necessary because of the closing up of the erupting space of the permanent lateral incisors, often due to the premature extraction or loss of the deciduous lateral incisors.

Premature loss of the deciduous teeth in this manner should be provided for by the mechanical retention of the space necessary for the eruption of the permanent successors, otherwise the dental arch will contract in the spaces of these teeth, leaving no room for their eruption. The banding of the deciduous cuspids, and connecting these bands by a lingually soldered retaining wire, will prevent the malocclusion from developing, and is an operation that any dentist can easily perform.

In the event of the premature loss of a molar in the deciduous arch, as occasionally happens through necessary extraction, it is always advisable to guard against the subsequent contraction of the dental arch by holding the space of the lost tooth open by means of bands on the teeth on either side of the space connected by a lingual wire.

The prolonged retention of deciduous teeth works almost as much havoc in the developing permanent arch as the premature loss of these teeth, and the mouths of children should be carefully watched for such abnormal conditions. The most common instance of this condition is observed in the retention of the lower deciduous central incisors beyond the time for their natural absorption, their roots being unabsorbed and deflecting the erupting permanent centrals lingually. If there is room for the permanent incisors to erupt, the only operation necessary is the extraction of the deciduous centrals when the permanent centrals will of their own volition grow forward into the space.

The prolonged retention of the deciduous lateral incisors often deflects the crowns of the permanent laterals lingually, indicating the necessity for the immediate extraction of the deciduous lateral incisors, and possibly the slight expansion of the dental arches to obtain sufficient room for the eruption of the permanent lateral incisors into position.

The prolonged retention of the deciduous cuspids or of the first or second deciduous molars, when there is no evidence of the approaching eruption of the permanent successors, is a matter of some concern, since the germ of the succeeding permanent tooth is occasionally absent, and the extraction of one of these teeth without an x-ray diagnosis, showing the presence of the succeeding permanent tooth, would be questionable practice. In the absence of a permanent successor, as shown by the x-ray, the deciduous cuspid or molar usually does not suffer from the absorption of its root, and may do good service for a lifetime if properly cared for.

A case in point is one in which the dentist consulted me as to the advisability of extracting the deciduous second molars, they having been retained beyond the period of their natural loss. I made a radiograph of both lateral halves of the upper dental arch, and finding the permanent second bicuspids missing, advised the preservation of the deciduous second molars, and they have done good service for many years since the advice was given.

Supernumerary teeth are not infrequently the causes of malocclusion, which the dentist can prevent by the extraction of these teeth before they can cause a deflection or transposition of the permanent teeth.

Occasionally a supernumerary tooth will erupt alongside the normal tooth, which it will very closely resemble, and, as in a case, in which the normal lateral and the supernumerary lateral are so much alike in the form of their crowns it is necessary to make a radiogram of their roots to determine which one to extract and which one to preserve.

The extraction of the first permanent molar is a common cause of malocclusion, for the teeth on either side of the space of the lost first molar drift into the space, and the dental arches collapse, causing malocclusion, in the same manner that an arch of masonry collapses when the keystone is removed. If I could leave with you one thought which I would wish to impress more than another it is this—*Guard the first permanent molar from the ravages of caries from the moment of its eruption until it is safe from caries in the sulci and from caries caused by contact with the too often carious distal surface of the second deciduous molar.* It has been my experience that altogether too many first permanent molars have been lost, with consequent serious malocclusions resulting because of the lack of frequent dental care of these teeth.

Now, having pointed out a number of the so-called local causative factors of malocclusion, and suggested methods of prevention of serious malocclusion resulting therefrom, I wish to say a few words as to the management of children in office practice. It might be asked why it is that the dentist often dreads the necessary visits of children, while the orthodontist looks forward to their coming with pleasure. The reason for this is probably because the dentist's work is associated with more or less pain, while the orthodontist is able, through the use of delicately constructed and skillfully applied appliances, to avoid pain. If it were possible for the dentist to fill the cavities of deciduous teeth without any more pain than the orthodontist causes in fitting bands to the teeth, the children would not be so averse to the dental chair, and the dentist would take more pleasure in his work for the children.

There is a difference in the way different dentists handle children also; in a community one or two dentists will get a reputation for the skillful handling of children, due, no doubt, to their love for or better understanding of children, and for their avoidance of pain in caring for their teeth.

Fear, if present, should be banished at the first visit of the child, and, once banished, it should never be allowed to return through any rough handling or carelessness on the part of the operator. Tears are always near the surface in children, and are always the signal for an immediate armistice and cessation of hostilities as it were, when the cause of the tears may be discovered, and by means of a diversion of a frivolous nature, equilibrium of emotions is once more restored, and the work finished with a careful avoidance of the incident which caused the tears.

It is all a matter of psychology in dealing with children; their minds are like an open book, very impressionable, ready for suggestions of a nature which will interest and please them, and if their thoughts are directed in the right channel they will forget that they are in a dental office, and will fill the office with their sunshine and laughter, and often drive away a fit of the blues.

Can you imagine a child with a mouth full of a plaster impression laughing until the tears ran down its cheeks? That happened in my office this last week, and was a pleasant diversion, although an impression need never cause any fear or disagreeable sensations if carefully and scientifically done.

Find out what is occupying the child's interest at the time, and converse with it on that subject, adding the ideas which it is looking for, stimulating its enthusiasm, thus proving to them that you are deserving of their confidence, and in the meantime, gaining their friendship.

Often a child will come in with a big idea; help him along with it. He will forget all about the office and everything that might be disagreeable about it in a few minutes, and your work will go along smoothly to its completion without a ripple to mar the surface. To illustrate, one of my little patients, a boy who usually has to be constantly watched because of his fidgety actions and boisterous behavior, announced to me when he came in the other day that he was going to be an inventor, his father having bought him a bench with a number of tools to work with. He and his companions had formed an inventor's club, and were going to invent things and make some money on them. I immediately encouraged the idea, showed him some inventions of my own, and finding that he did not know what to invent first, suggested to him that I needed a small white enamel waste receiver attached to the cabinet, to be worked with a foot lever much as a surgeon's waste receiver is operated.

He fell for the idea at once, saying that his mother had an old flour can which he could use to start with, and when they got things going they would give me one, I having told him of the possible great demand for them by the dental trade, and of the possibility of making considerable money in selling them. We have established a relationship of camaraderie, based on business presumably, which will probably last until I can complete his case, but giving me a control over his actions which I could not so easily obtain any other way.

Suggest to the children the subject of conversation while in the office, and, if you are keen, you will find a way to interest them along lines they unconsciously suggest to you.

The mysterious always appeals to them, and a few sleight-of-hand tricks will not come amiss. Lighting the gas with a pencil attached to the electric lighting current never fails to elicit their interest and admiration. Pumping up the chair or running the dental engine often serves a similar purpose.

Stories have a remarkable effect, and it is well to keep a stock of them on hand for purposes of diversion. The office magazines should be chosen with the idea of interesting the children rather than the adult entirely. Even the furniture may be selected with a view to their needs, especially in an orthodontist's office.

It must be remembered that the child lives in a little world of its own, a world of effervescent joy and happiness, a combined mixture of birthdays, Christmas and other holidays, bed-time stories, candy, cake and ice cream, picnics and games.—*Oral Health,* and *International Journal of Orthodontia.*

